# Urine cultures and antibiotics for urinary tract infections in Dutch general practice

**DOI:** 10.1017/S146342361800066X

**Published:** 2018-08-31

**Authors:** Karlijn M.J. Ganzeboom, Annemarie A. Uijen, Doreth T.A.M. Teunissen, Willem. J.J. Assendelft, Hans J.G. Peters, Jeannine L.A. Hautvast, Cornelia H.M. Van Jaarsveld

**Affiliations:** 1 Medical Student, Department of Primary and Community Care, Radboud University Medical Centre, Nijmegen, The Netherlands; 2 Coordinator Famely Medicine Network, General Practitioner, Department of Primary and Community Care, Radboud University Medical Centre, Nijmegen, The Netherlands; 3 General Practitioner, Senior Researcher, Department of Primary and Community Care, Radboud University Medical Centre, Nijmegen, The Netherlands; 4 Professor in Primary Care, Head of Department, Department of Primary and Community Care, Radboud University Medical Centre, Nijmegen, The Netherlands; 5 Datamanager, Department of Primary and Community Care, Radboud University Medical Centre, Nijmegen, The Netherlands; 6 Senior Researcher in Infectious Diseases, Department of Primary and Community Care, Radboud University Medical Centre, Nijmegen, The Netherlands; 7 Senior Epidemiologist, Department of Primary and Community Care, Radboud University Medical Centre, Nijmegen, The Netherlands

**Keywords:** antibacterial agents, cultures, drug resistance, general practice, microbial, urinary tract infections

## Abstract

**Background:**

Urinary tract infections (UTIs) are common in general practice, and antibiotic resistance is often seen. Urine cultures are advised by the Dutch national UTI guideline for patients at high risk of UTI complications. Prudent use of antibiotics and taking into account national guidelines and urine culture results are important to combat antibiotic resistance in general practice.

**Aim:**

To identify subgroups of UTI patients in which the use of urine cultures and antibiotic prescriptions deviates from the national guidelines.

**Methods:**

We investigated associations of several characteristics with urine culture orders in patients with UTI in 2015 from seven Dutch general practices (*n*=1295). These included subgroups at risk for UTI complications, comorbidities, age and history of UTI recurrence. In addition, we assessed the level of adherence to the guideline for antibiotic prescriptions in subgroups at risks for UTI complications.

**Findings:**

Urine cultures were ordered in 17% (*n*=221) of patients, more frequently in high-risk patients (32%) than in low-risk patients (7%), for UTI complications (OR=6.4; 95% CI 4.6–9.0). In low-risk patients, 91% received antibiotics that were recommended in the guideline. For high-risk patients this percentage ranged widely, and was particularly low in the risk groups with signs of tissue invasion (29–50%). Diagnostic and therapeutic adequacy can still be improved by increasing the adherence to the guideline in UTI patients at high risk for complications. This may contribute to containing antibiotic resistance in UTI by ordering urine cultures and use the results to adjust prescriptions to antibiotic susceptibility of the uropathogen.

## Introduction

Resistance to antibiotics is a worldwide problem, which may be reduced by prescribing less antibiotics and reducing inappropriate prescriptions (Wise *et al.*, 1998; Butler *et al.*, 2007). More than 90% of antibiotics in Europe are prescribed in primary care (Goossens *et al.*, 2005). For urinary tract infections (UTIs) the initial prescription of antibiotics is usually ‘blindfolded’, without knowing the causing uropathogen and its susceptibility to antibiotics. This is considered sufficient for healthy non-pregnant women, because of the high prevalence of uropathogen *Escherichia coli* and its susceptibility to nitrofurantoin and fosfomycin (Den Heijer *et al.*, 2010). However, there are several groups of patients, including pregnant women and all men, who are at high risk for UTI complications (pyelonephritis, prostatitis or urosepsis) because of different uropathogens or immunosuppression. Therefore, the guideline of Dutch College of General Practitioners (GPs) on UTI (NHG-guideline) (Van Pinxteren *et al.*, 2013) recommends a urine culture and specific antibiotics for these high-risk patients. If a culture is ordered, the guideline recommends that antibiotics should be started empirically in anticipation of the culture result, after which a switch can be made if resistance is seen. This Dutch guideline is very similar to the UK guideline for GPs (Health Protection Agency, 2011), with the exception of two risk factors: recurrent UTI is not considered a risk factor for complications in the Dutch guideline, whereas, on the other hand, immune disorders are not mentioned in the UK guideline (Health Protection Agency, 2011). The two guidelines are quite similar regarding antibiotic recommendations. Both guidelines advise nitrofurantoin and trimethoprim as first choice antibiotics for low-risk patients, however, the Dutch guideline also includes fosfomycin. In addition, in the Dutch guideline co-amoxiclav is the second choice instead of amoxicillin for any patient without tissue invasion and co-trimoxazole for patients with tissue invasion.

In Dutch general practice, resistance to the recommended antibiotics varied from 2% (nitrofurantoin) to 26% (trimethoprim) for the most common uropathogen *E. coli* in cultures (De Greeff *et al.*, 2015).

In most countries, studies mainly focus on treatment of UTI and rarely on use of cultures in general practice. A Spanish GP study shows that for 33% of low-risk patients a culture is ordered, even though not meeting the national guideline criteria for a culture (Llor *et al.*, 2011). A study among GPs in the UK shows a culture was ordered in 40% of low-risk patients and in 39–98% of high-risk patients, depending on risk factor.

When prescribing antibiotics for UTI, only 42% (Braspenning *et al.*, 2004) to 50% (Van Bergeijk and Berger, 2008) of Dutch GPs appear to adhere to the therapeutic recommendations in the national guideline. The current guideline on UTI is well-known among GPs in The Netherlands, but a study also showed that Dutch GPs still perceive several barriers implementing this guideline, like unavailable diagnostic materials or unavailable dosages of medication (Lugtenberg *et al.*, 2010).

To support a more rational prescription of antibiotics in primary care, more data are needed on current use of cultures and related to this, antibiotic prescriptions. Therefore, our aim is to identify subgroups of UTI patients in which the use of urine cultures and antibiotic prescriptions deviates from the guidelines. We also investigate the influence of established risk factors for UTI complications (eg pregnancy, immunodeficiency), common comorbidities (like cardiovascular or pulmonary diseases), age and a history of recurrent UTI on urine culture requests.

## Methods

This is an observational study using anonymous electronic patient records (EPR) from seven practices (24 GPs and 30,452 patients) from the Family Medicine Network (FaMe-Net). This is a practice-based research network from the Radboud University Medical Centre in Nijmegen, The Netherlands. FaMe-Net originates from a fusion between the Continuous Morbidity Registration (Van Weel, 2008) and Transitionproject (Okkes *et al.*, 2005). Of seven practices, three practices are located in Nijmegen (Southeast; urban and semi-urban area), two practices in Amstelveen (West; semi-urban area), one in Olst (East; rural area) and one in Franeker (North; rural area). The study practices are representative to Dutch practices in terms of average patient population size, male-to-female GP ratio and proportion of practices that include GP trainees (CBS Statistics Netherlands; Netherlands Institute for Health Services Research (NIVEL)). However, the majority of our study practices are group practices consisting of three or more GPs, whereas fewer solo or duo practices are included in comparison to the general practices across The Netherlands. The patient population in this network is representative of the general Dutch population in terms of age and sex (CBS Statistics Netherlands). Participating GPs have special interest in primary care research and code each episode of care according to the International Classification of Primary Care (ICPC) (Hofmans-Okkes and Lamberts, 1996). An episode of care is defined as a health problem in an individual from the first to the last visit related to the specified health problem. All actions from the GP, including physical examination, diagnostic tests and prescriptions, are systematically coded. The validity of registration is high, as participating GPs meet regularly to discuss registration and diagnostic criteria. Moreover, the system warns the GP in case of error or inconsistency in registration. Research with FaMe-Net data is exempted from ethical review by the CCMO (Dutch Central Committee on research involving human subjects).

The study population included all patients with ICPC codes U71 (cystitis), U70 (pyelonephritis) or Y73 (prostatitis) that presented to their GP in 2015 (*n*=1295). If patients had multiple UTIs, only the first diagnosis in 2015 was included.

### Definition of risk for UTI complication

The risk of UTI complications was classified according to the Dutch national guideline on UTI and is summarized in Table 1 (Van Pinxteren *et al.*, 2013). High-risk patients had at least one of the risk factors. Consequently, low-risk patients include all patients with an UTI without any of these high-risk criteria.

**Table 1:** Characteristics of study population and likelihood of urine culture request in subgroups (*n*=1295)

	*n*	(%)	Cultures / *n* (%)	Unadjusted OR^c^ (95% CI)	Adjusted OR^d^ (95% CI)
Total	1295	(100)	221/1295 (17.1)	−	−
Low-risk for complications	765	(59.1)	52/765 (6.8)	−	−
High-risk (>1 risk factor)^a^	530	(40.9)	169/530 (31.9)	**6.4 (4.6–9.0)**	−
Male	175	(13.5)	69/175 (39.4)	**4.1 (2.9–5.9)**	**6.2 (4.0–9.6)**
Symptoms of tissue invasion	163	(12.6)	54/163 (33.1)	**2.9 (2.0–4.1)**	**1.7 (1.1–2.6)**
Immune disorder	153	(11.8)	27/153 (17.6)	1.0 (0.7**–**1.6)	0.9 (0.5**–**1.6)
Renal/bladder disease	142	(11.0)	35/142 (24.6)	**1.7 (1.1–2.6)**	1.4 (0.9**–**2.3)
Age <12 years	70	(5.4)	39/70 (55.7)	**7.2 (4.4–11.9)**	**9.9 (5.4–17.8)**
Pregnancy	45	(3.5)	23/45 (51.1)	**5.6 (3.0–10.1)**	**12.8 (6.3–26.1)**
Indwelling catheter	30	(2.3)	8/30 (26.7)	1.8 (0.8**–**4.1)	0.7 (0.3**–**1.8)
Antibiotic prophylaxis	4	(0.3)	3/4 (75.0)	**14.8 (1.5–142)**	**31.6 (3.0–332)**
Comorbidity (≥1 present)	485	(37.5)			
Cardiovascular disease	263	(20.3)	37/263 (14.1)	0.8 (0.5–1.1)	0.6 (0.3–1.1)
Pulmonary disease	196	(15.1)	27/196 (13.8)	0.7 (0.5–1.2)	0.8 (0.5–1.3)
Thyroid dysfunction	80	(6.2)	12/80 (15.0)	0.8 (0.5–1.6)	1.4 (0.7–2.8)
Nervous system	30	(2.3)	6/30 (20.0)	1.2 (0.5–3.0)	0.5 (0.2–1.6)
Rheumatoid arthritis	27	(2.1)	4/27 (14.8)	0.8 (0.3–2.5)	1.6 (0.5–4.9)
Dementia	22	(1.7)	7/22 (31.8)	2.3 (0.9–5.7)	**3.9 (1.3–11.4)**
Inflammatory bowel	18	(1.4)	4/18 (22.2)	1.4 (0.5–4.3)	1.5 (0.3–4.7)
Recurrent UTI	99	(7.6)	21/99 (21.2)	1.3 (0.8–2.2)	**1.9 (1.1–3.4)**
Age (years)^b^					
12–18	36	(2.8)	8/36 (22.2)	1.6 (0.7–3.7)	2.1 (0.9–5.2)
19–24	97	(7.5)	8/97 (8.2)	0.5 (0.2–1.1)	0.7 (0.3–1.6)
25–44	332	(25.6)	51/332 (15.4)	1.0 (0.7–1.6)	0.8 (0.5–1.3)
45–64	366	(28.3)	55/366 (15.0)	1.0	1.0
65–74	155	(12.0)	21/155 (13.5)	0.9 (0.5–1.5)	0.9 (0.5–1.5)
>75	239	(18.5)	39/239 (16.3)	1.1 (0.7–1.7)	1.0 (0.5–1.8)

a Patients with multiple risk factors are included for each risk factor.

b For age 0–12 months, see risk factor ‘Age <12 years’.

c Statistically significant results are printed in bold. OR present the likelihood of a urine culture being requested in patients with that characteristic compared with all other patients without that specific characteristic.

d OR are adjusted: models include all risk factors, all comorbidities, a history of recurrent UTI and age groups compared with the largest age group of 45–64 years simultaneously.

### Data extraction

Data from the EPR were extracted in two steps. In step one, a data set including all ICPC codes registered in 2015 were extracted to identify patients that presented with a UTI to their GP in 2015. In step two, complete data from all available years before 2015 were extracted for all selected UTI patients. Data were extracted from the EPR, which contains ICPC codes (diagnosis), Anatomical Therapeutic Chemical (ATC) codes (medication prescriptions using Anatomical Therapeutic Chemical classification system), intervention codes, laboratory codes, written lines by the GP and letters from other (medical) professions. For each subject the characteristics that matched the criteria for a risk group were classified, using a combined list of ICPC, ATC and laboratory codes and keywords (see Appendices I, II and III for full list).

### Urine cultures

We extracted data on all urine cultures ordered one day before UTI diagnosis until 14 days after, by an electronic search of the written lines in the EPR and by screening the letters from microbiologists and laboratories.

### Medical history

We retrieved data on common comorbidities (other than risk factors for complications of UTI) with a list of ICPC codes (see Appendix II) using full medical history data in the EPR. Comorbidities included cardiovascular and pulmonary disease, inflammatory bowel diseases, thyroid dysfunctions, rheumatoid arthritis, dementia and diseases of the nervous system.

Recurrent UTI was defined as three or more UTIs in the preceding 12 months (Epp *et al.*, 2010).

### Statistical analysis

The main outcome was the order of a culture, and the main determinants were subgroups at high risk for complications of UTI, comorbidities, history of recurrent UTI and age groups compared with the largest age group of 45–64 years. We categorized age of patients into seven groups for analyses. We analysed the association between urine culture orders and each of the above-described patient characteristics individually with an unadjusted logistic regression analysis. An adjusted logistic regression model, where all the above-described characteristics were entered simultaneously, was used to identify which factors contributed independently. We evaluated the compliance of GPs to the national guideline (Van Pinxteren *et al.*, 2013) for the use of urine cultures and the type of prescribed antibiotics with descriptive statistics and χ^2^-tests. All analyses were performed using SPSS version 22. A *P*-value of <0.05 was considered statistically significant.

## Results

### Patient characteristics

In total, 530 (40.9%) patients had one or more risk factors for complications of UTI (Table 1). The most common high-risk characteristic was male gender (13.5%), followed by symptoms of tissue invasion (12.6%), immune disorders (11.8%) and renal/bladder diseases (11.0%). Selected comorbidities were present in 37.5% of patients, and 7.6% of patients had a history of recurrent UTI.

### Urine cultures

A urine culture was ordered in 221 patients (17.1%) (Table 1). Urine cultures were more commonly ordered in high-risk (31.9%) than low-risk patients (6.8%) (OR=6.4, 95% CI 4.6–9.0). Relatively more cultures were ordered in patients that used antibiotic prophylaxis (75.0%), followed by patients aged under 12 (55.7%), and pregnant women (51.1%), although fewer cultures were ordered in patients with an indwelling catheter (26.7%), renal/bladder disease (24.6%) or immune disorder (17.6%).

As expected, urine culture orders were more common among several subgroups of patients at high risk for complications. Adjusted analyses (including all risk factors for complications, comorbidities, a history of recurrent UTI and age) show that urine cultures were significantly more often ordered in patients with antibiotic prophylaxis (OR=31.6), pregnancy (OR=12.8), age <12 years (OR=9.9), male (OR=6.2) or symptoms of tissue invasion (OR=1.7) compared with patient groups without these characteristics, respectively (see Table 1). Strikingly, urine cultures were not more often ordered in patients with immune disorder, renal/bladder disease or an indwelling catheter, whereas these patients are also at high risk for complications of UTI, and the national guideline recommends urine cultures in these patients. In addition, culture orders were significantly more common in patients with recurrent UTI (OR=1.9) and dementia (OR=3.9), compared with those without recurrent UTI or dementia, respectively. There was no statistically significant influence of age or other comorbidities on urine culture orders.

In 6.8% of low-risk patients, a urine culture was ordered. In the low-risk group urine cultures were more common in patients with dementia (OR=8.3; 95% CI 1.8–38.3, results not shown). None of the other comorbidities, age or recurrent UTI had a statistically significant effect on urine culture orders.

Among high-risk patients, we found no influence of comorbidities, age or recurrent UTI on urine culture orders (results not shown).

### Antibiotics

Almost all UTI patients (94%) were prescribed antibiotics, mostly nitrofurantoin (68.7%), followed by ciprofloxacin (8.8%) and fosfomycin (6.5%). In the low-risk group, 31 (4.1%) patients did not receive antibiotics, compared with 47 (8.9%) in the high-risk group (*P*-value <0.001). As shown in Table 2, recommended antibiotics (first, second or third choice in the guideline) were prescribed to 91.4% of low-risk patients. For high-risk patients adherence to the recommended antibiotics varied strongly by risk factor. Higher adherence was seen in children and pregnant women without tissue invasion, 91.7 and 80.0%, respectively. Lower adherence was observed in men without tissue invasion (50%) and high-risk (non-pregnant female) patients with tissue invasion (range 28.6–50.0%) (see Table 2).

**Table 2:** Antibiotic prescriptions for UTI in subgroups at risk for UTI complications, compared with recommended prescriptions in the Dutch GP guideline (Van Pinxteren *et al.*, 2013)

	Antibiotic prescription, *n* (%)				
	According to guideline	First choice^c^	Second/third choice^d^	Not according to guideline	Not prescribed
Low-risk for complications, *n*=765	699 (91.4)	618 (80.8)	81 (10.5)	35 (4.6)	31 (4.1)
High-risk, *n*=530^a^					
Children (age <12 years)					
No tissue invasion, n=48	44 (91.7)	38 (79.2)	6 (12.5)	1 (2.1)	3 (6.3)
With tissue invasion, n=20	13 (65.0)	12 (60.0)	1 (5.0)	5 (25.0)	2 (10.0)
Pregnant					
No tissue invasion, *n*=40	32 (80.0)	27 (67.5)	5 (12.5)	1 (2.5)	7 (17.5)
With tissue invasion, *n*=5	Unknown ^e^	Unknown	0	3 (60.0)	2 (40.0)
Non-pregnant, aged >12 years^b^					
Men					
No tissue invasion, *n*=114	57 (50.0)	52 (45.6)	5 (4.4)	48 (42.0)	13 (11.4)
With tissue invasion, *n*=55	43 (78.2)	32 (58.2)	11 (20.0)	7 (12.7)	5 (9.1)
Immune disorder					
No tissue invasion, *n*=13	8 (61.5)	8 (61.5)	0	4 (30.8)	1 (7.7)
With tissue invasion, *n*=8	3 (37.5)	3 (37.5)	0	3 (37.5)	2 (25.0)
Indwelling catheter					
No tissue invasion, *n*=21	8 (38.1)	8 (38.1)	0	12 (57.1)	1 (4.8)
With tissue invasion, *n*=7	2 (28.6)	1 (14.3)	1 (14.3)	3 (42.9)	2 (28.6)
Renal/bladder disease					
No tissue invasion, *n*=120	72 (60.0)	62 (51.7)	10 (8.3)	39 (32.5)	9 (7.5)
With tissue invasion, *n*=20	10 (50.0)	8 (40.0)	2 (10.0)	6 (30.0)	4 (20.0)
Antibiotic prophylaxis					
No tissue invasion, *n*=4	4 (100.0) ^f^	0	0	0	0
With tissue invasion, *n*=0	–	–	–	–	–
Other					
No tissue invasion, *n*=0	–	–	–	–	–
With tissue invasion, *n*=64	23 (35.9)	15 (23.4)	8 (12.5)	41 (64.0)	0

(NHG=Nederlands Huisartsen Genootschap/Dutch College of General Practitioners)

a For each high-risk group, patients with and without tissue invasion are distinguished because of different antibiotic recommendations in NHG-guideline.

b Patients with multiple risk factors are included for each risk factor.

c First choice antibiotic is nitrofurantoin for all subgroups, except for patients with tissue invasion where first choice is co-amoxiclav in children aged <12 years, and ciprofloxacin in other patients with tissue invasion. Pregnant women with tissue invasion are recommended to be referred to hospital.

d Second choice antibiotic is fosfomycin (low-risk patients), co-amoxiclav (children without tissue invasion/pregnant women without tissue invasion/non-pregnant patients aged >12 years with tissue invasion), co-trimoxazole (children with tissue invasion), trimethoprim (non-pregnant patients aged >12 years without tissue invasion); third choice antibiotic is only listed for low-risk patients (ie trimethoprim) and non-pregnant patients aged >12 years with tissue invasion (ie co-trimoxazole).

e There were no data available due to referrals from hospital (as recommended in guideline).

f National GP guideline advises a switch in antibiotics.

## Discussion

### Most important findings

This study shows a low percentage (32%) of urine culture ordered in patients with risk factors for complications of UTI. Culture orders were particularly low in patients with immune disorder (18%), renal/bladder disease (25%) or an indwelling catheter (27%). Antibiotics were prescribed to the majority of patients (94%), but compliance to the national guideline varied broadly by risk factor for complications (29–92%).

### Strengths and limitations

One of the major strengths is the high-quality of recording in this cohort, as inconsistency in registration is flagged by the system and GPs from the network discuss registration issues regularly. Also, the number of patients with an UTI included in our study was high (*n*=1295) and we had access to individual patient data from the EPR. This is the first study in The Netherlands that examined urine culture orders for UTI in general practice, identifying patient characteristics in whom a culture was more frequently ordered.

There are several limitations worth mentioning. First, the time period in which temporary risk factors (pregnancy and indwelling catheter) were present had to be estimated. Therefore, we compared the prevalence of these risk factors with those found in the literature, and were found to correspond to our figures. Van Bergeijk and Berger (2008) found 3.1% pregnant patients with UTI in Dutch general practice (compared with 3.5% in our study). Indwelling catheter appeared in 3% of female patients in the study of Hummers-Pradier *et al.* (2005), compared with 2.3% in our study. For indwelling catheters, we assumed a standard use of 12 weeks. Patients who had their catheter removed sooner are potentially misclassified as high risk, which may (partly) explain the lower rate of urine culture orders in this group. Second, it was not possible to identify patients with ‘failure of two blindfolded antibiotic prescriptions’, which is listed as an additional risk factor for complications of UTI in the national guideline, because we were not able to distinguish in the EPR if symptoms had disappeared or continued between two prescriptions for antibiotics. Third, the result of slightly more patients in the high-risk group that did not receive antibiotics (8.9% compared with 4.1% in the low-risk group) was unexpected. This finding might be partially explained, by having misclassified some patients as high risk (ie not all patients with malignancies received chemotherapy/radiotherapy, as we have assumed). Also, some high-risk patients may have been referred to hospital and were prescribed antibiotics by a medical specialist, which is not registered in the EPR in general practice. Lastly, the GPs in our database were all part of the FaMe-Net practices, which are GPs with special interest in research and have frequent meetings to optimize accurate registration in the EPR, so perhaps the compliance with the guideline is higher than among other GPs.

### Comparison to other literature

In comparison with the study by Llor *et al.* (2011) in Spain, who found that GPs ordered urine cultures in 33% of low-risk patients, the GPs in our study performed better by ordering fewer cultures in low-risk patients (7%). This might be because of the absence of dipslides in the Spanish study population, which means the only available diagnostic tool is a culture.

Ironmonger *et al.* (2016) questioned GPs in the UK on the use of urine culture in patients with and without risk factors. As we did, they found a notable difference in culture orders in low-risk (40%) and high-risk patients, with differences by risk factor (38% for indwelling catheter to 98% for male patients). However, they investigated only a selection of risk factors in five hypothetical patients, making it difficult to compare the reported percentages with our data. In comparison with a Dutch study by Den Heijer *et al.* (2010), who investigated the antibiotic prescription rate by GPs for low-risk patients with UTI and the susceptibility of *E. coli* to common antibiotics, the percentage of antibiotic prescriptions corresponding to the guideline for low-risk patients in our study is higher, namely 91% compared with 77%. This might be partially due to the continuing decrease in use of quinolones for low-risk patients, as has been advised in the national guideline since 2005.

### Implications

Both diagnostic and therapeutic adequacy could be improved, which is implicated by the relatively low percentage of culture orders and high use of inappropriate antibiotics (ie non-recommended in the guideline) in several subgroups of high-risk patients. This is worrying because resistance to commonly prescribed antibiotics in the high-risk group is often seen (De Greeff *et al.*, 2015). Not ordering a urine culture in high-risk patients can have several reasons, for example, the GP is not aware of all risk factors for complications of UTI, the GP might not see the benefit of a culture owing to experience and the low incidence of complications, or possibly the patient does not want a culture because of financial costs. The underlying motives for not ordering cultures should be further explored.

The percentage of culture orders in low-risk patients is relatively low (7%), but not 0% as would have been expected with perfect compliance to guidelines. This group might still contain some high-risk patients with risk factor ‘failure of two blindfolded given antibiotics’, which we have not been able to identify. Furthermore, we found that patients with dementia are more likely to get a culture order. In case of an unexplained fever in patients with dementia, a urine culture might be routinely performed to find the cause. Moreover, owing to a difficult anamnesis, the efficacy of antibiotics is hard to monitor in these patients, so a urine culture may contribute in evaluating treatment effects. As dementia is not specifically mentioned in the guideline, it would be of interest to investigate the reasons of GPs for performing more cultures in this patient group, and its potential value.

For high-risk patients the adherence to recommended antibiotics varied strongly by risk factor. Reasons for non-adherence should be further explored, focussing both on GP-related factors and patient influences. It is unknown whether the lower rates of adherence in some subgroups of patients are based on intentional or non-intentional decisions by GPs or driven by patient requests.

The Dutch GP guideline on UTI is currently under revision, and our findings imply the need of critical review of current policies, particularly in the highlighted subgroups. As non-adherence is low in subgroups of patients, future revisions of the guideline should evaluate current policies and include information on the cost-effectiveness of urine cultures in specific subgroups of patients and address issues related to non-adherence.

In conclusion, in The Netherlands urine cultures are ordered by GPs in only a third of patients at high risk for complications of UTI, even though the guideline recommends cultures in all high-risk patients. Antibiotics were prescribed to nearly all patients, but compliance to the national guideline varied a lot by risk factor. Diagnostic and therapeutic adequacy can be improved in UTI patients at high risk for complications by ordering more cultures and adjust prescriptions to antibiotic susceptibility of the uropathogen, and improve antibiotic prescriptions adhering to the national guideline. This may contribute to containing antibiotic resistance in UTI.

## Appendix I: Additional information on methods

### Full definition for immunocompromised patients

Immune disorders include diabetes mellitus, the use of chemotherapy or radiotherapy (defined as a malignancy in the previous two years* for which those are commonly received), use of immunosuppressants or immunostimulants, and a medical history with immune deficiencies, AIDS/HIV, splenectomy or a received organ transplantation.

*According to the Health Council of The Netherlands (Van Pinxteren *et al.*, 2013), most of the treatment and short-term follow-up with specialized care in the hospital takes place within the first and second year after diagnosis of a malignancy. Therefore, we consider patients within the first two years after diagnosis of a malignancy for which chemotherapy or radiotherapy is often given, to be immunocompromised, owing to the high chance of receiving either treatments.

### Electronic search with keywords

Risk factors, comorbidities and diagnostic tests in the written lines of the EPR appeared in the database as free text and were searched using the following protocol to optimize standardized data extraction. The free text was searched electronically for keywords, with the use of iterative processing (ie including additional keywords based on interim checks) to fine tune the result. To find the absence or presence of a symptom or test, different positive and negative prefixes and suffixes were used in addition to searches with the single keywords. Spelling errors in keywords were anticipated and overcome by testing possible errors. The final result of the electronic search was checked with random samples and proved to be of good quality.

### Classifying diabetes

In the NHG-guideline, it is described that otherwise healthy women with diabetes may be considered as low risk for complications of UTI. After selection of all female patients with diabetes, but with no other comorbidities, no risk factor and aged under 60 (Health Protection Agency, 2011), we identified only three patients and we have therefore chosen to classify all diabetic patients as high risk.

### Other

- The date of all given ICPC and ATC codes for risk factors and comorbidities had to occur before the date of the UTI of that patient, in order to be included in the analysis.
- All patients had to be subscribed to the practice for the full period of data collection.
- In case a patient had a culture found in multiple ways, the most extensive or detailed one was used.

## Appendix II: ICPC codes used to identify comorbidities

**Table:**

Comorbidity	ICPC code	Description
Cardiovascular disease	K74	Ischaemic heart disease with angina
	K75	Acute myocardial infarction
	K76	Ischaemic heart disease without angina
	K77	Heart failure
	K78	Atrial fibrillation/flutter
	K80	Cardiac arrhythmia NOS
	K82	Pulmonary heart disease
	K83	Heart valve disease NOS
	K84	Heart disease other
	K89	Transient cerebral ischaemia
	K90	Stroke/cerebrovascular accident
	K91	Cerebrovascular disease
	K93	Pulmonary embolism
Pulmonary disease	R95	Chronic obstructive pulmonary disease
	R96	Asthma
Inflammatory bowel diseases	D94	Chronic enteritis/ulcerative colitis
Thyroid dysfunctions	T85	Hyperthyroidism/thyrotoxicosis
	T86	Hypothyroidism/myxoedema
Rheumatoid arthritis	L88	Rheumatoid/seropositive arthritis
Dementia and diseases of the nervous system	N86	Multiple sclerosis
	N87	Parkinsonism
	N88	Epilepsy
	P70	Dementia

NOS = Not Otherwise Specified

## Appendix III: Definitions of risk factors for complications of UTI, according to the NHG-guideline and description of data extraction

**Table:**

Risk factor	Definition	Data extraction
Male gender		General information from EPR
Symptoms of tissue invasion	Fever, chills, malaise, flank or perineal pain, acute confusion or delirium	Laboratory codes^a^ for patient’s temperature were used and the written lines in the EPR were electronically searched for keywords^a^ on symptoms of tissue invasion. Also, ICPC U71 pyelonephritis, Y73 acute prostatitis and urosepsis were used
Immune disorders	Including diabetes mellitus, immunosuppressant drugs, medical history with immune disorders and malignancies in the past two years with high change of receiving chemotherapy/radiotherapy	The use of immunosuppressant/stimulant medication was based on ATC codes in EPR. Time period of usage was calculated with dosage per day and amount of prescribed medication. A list of ICPC codes^a^ was used for immune disorders and malignancies
Renal/bladder disease	Including severe renal insufficiency (GFR<30), renal cysts, renal lithiasis, neurological bladder disorders, urinary retention, prostatism	A list of ICPC codes^a^ was used for renal/bladder diseases, as well as the most recent GFR before the UTI
Age <12 years		General information from EPR
Pregnancy	In any trimester	A list of ICPC codes^a^ was used. If an ICPC delivery code was present, the estimated time of pregnancy was 280 days before date of delivery. If only a pregnancy code was present, it was assumed that women presented to their GP for the first time four weeks into pregnancy
Indwelling catheter	Presence of indwelling urinary catheter	Both an electronic search of written lines in EPR for keyword ‘catheter’ was performed and the EPR prescription list was used. For each prescribed catheter on the medication list 12 weeks (maximum time period for one catheter) were added to the date of prescription, to estimate the time period of presence
Antibiotic prophylaxis	Trimethoprim 50–100 mg or nitrofurantoin 50 mg once a day as prophylaxis for UTI	ATC codes^a^ in EPR were used. The time period during which prophylaxis was used was calculated based on the number of prescribed tablets
Failure of two ‘blindfolded’ prescribed antibiotics		It was not possible to reliably find the presence of this risk factor in EPR

(EPR=electronic patient record; ICPC code=International Classification of Primary Care code; ATC code=Anatomical Therapeutic Chemical classification code; GFR=glomerular filtration rate)

a A full list of ICPC, ATC, laboratory codes and keywords is available from corresponding the author.
